# The Sizes and Composition of HDL-Cholesterol Are Significantly Associated with Inflammation in Rheumatoid Arthritis Patients

**DOI:** 10.3390/ijms241310645

**Published:** 2023-06-26

**Authors:** Ching-Kun Chang, En-Pei Isabel Chiang, Kuang-Hsi Chang, Kuo-Tung Tang, Po-Ku Chen, Hei-Tung Yip, Chu-Huang Chen, Der-Yuan Chen

**Affiliations:** 1 Rheumatology and Immunology Center, China Medical University Hospital, Taichung 404, Taiwan; kun80445@gmail.com (C.-K.C.); pago99999@gmail.com (P.-K.C.); 2 Translational Medicine Laboratory, Rheumatology Research Center, China Medical University Hospital, Taichung 404, Taiwan; 3 College of Medicine, China Medical University, Taichung 404, Taiwan; fionyip0i0@gmail.com; 4 Department of Food Science and Biotechnology, National Chung Hsing University, Taichung 402, Taiwan; chiangisabel@nchu.edu.tw; 5 Innovation and Development Center of Sustainable Agriculture, National Chung Hsing University, Taichung 402, Taiwan; 6 Department of Medical Research, Tungs’ Taichung Metroharbor Hospital, Taichung 435, Taiwan; kuanghsichang@gmail.com; 7 Center for General Education, China Medical University, Taichung 404, Taiwan; 8 General Education Center, Jen-Teh Junior College of Medicine, Nursing and Management, Miaoli 356, Taiwan; 9 Division of Allergy, Immunology and Rheumatology, Taichung Veterans General Hospital, Taichung 407, Taiwan; crashbug1982@gmail.com; 10 Faculty of Medicine, National Yang Ming Chiao Tung University, Taipei 112, Taiwan; 11 Management Office for Health Data, China Medical University Hospital, Taichung 404, Taiwan; 12 Vascular and Medicinal Research, Texas Heart Institute, Houston, TX 77030, USA; cchen@texasheart.org; 13 Institute for Biomedical Sciences, Shinshu University, Nagano 390-8621, Japan; 14 College of Medicine, National Chung Hsing University, Taichung 402, Taiwan

**Keywords:** high-density lipoprotein (HDL), lipid metabolites, inflammation, anti-citrullinated peptide antibodies (ACPA), rheumatoid arthritis (RA)

## Abstract

Rheumatoid arthritis (RA), a chronic inflammatory disease, carries a significant burden of atherosclerotic cardiovascular diseases (ASCVD). With their heterogeneous composition, high-density lipoprotein (HDL) particles have varied athero-protective properties, and some may even increase ASCVD risk. In this prospective and cross-sectional study, we aimed to examine the relationship between HDL sizes/metabolites and inflammation in RA. Using ^1^H-NMR-based lipid/metabolomics, differential HDL-related metabolites were identified between RA patients and healthy control (HC) subjects and between RA patients with and without anti-citrullinated peptide antibodies (ACPA). The correlation between the discriminative HDL-related metabolites and C-reactive protein (CRP) was evaluated in RA patients. RA patients demonstrated higher particle number, lipids, cholesterol, cholesterol ester, free cholesterol, and phospholipids in large/very large-sized HDLs. ACPA-positive patients had higher L-HDL-C and L-HDL-CE but lower small-/medium-sized HDL-TG levels than ACPA-negative patients. An inverse correlation was found between CRP levels and small-sized HDLs. Janus kinase inhibitor treatment was associated with increased serum small-sized HDL-related metabolites and decreased CRP levels. We are the first to reveal the significant associations between RA inflammation and HDL sizes/metabolites. A potential link between ACPA positivity and changes in serum levels of HDL-related metabolites was also observed in RA patients.

## 1. Introduction

Low levels of high-density lipoprotein (HDL) cholesterol (HDL-c) are associated with high atherosclerotic cardiovascular disease (ASCVD) risk [1]. HDL is athero-protective through promoting cholesterol efflux and exerting its antioxidant, anti-apoptotic, and anti-inflammatory effects [2,3]. Navarro-Millán et al. revealed an inverse association of HDL-c levels with the risks of myocardial infarction and stroke in the predominantly older male rheumatoid arthritis (RA) cohort [4]. By using the liquid chromatography/mass spectrometry (LC/MS) approach, however, Camont et al. demonstrated the heterogeneity of HDL composition [5], which paralleled the variability in the key athero-protective effects of HDL-c. They also observed rich, small/dense, and protein-rich HDL3 particles in negatively charged phospholipids (PL), which are involved in cholesterol efflux and possess antioxidative and anti-inflammatory properties. Besides, chronic inflammation may be accompanied by multiple changes in HDL structure and thereby alter the athero-protective functions of HDL particles [6].

Rheumatoid arthritis (RA) is a chronic inflammatory articular disease complicated by a high ASCVD burden [7], which is probably due to the traditional risk factors, dyslipidemia, and systemic inflammation in this disease [8,9]. HDL-c can become dysfunctional after exposure to an inflammatory environment in chronic inflammatory diseases such as RA [10,11]. Charles-Schoeman et al. revealed that dysfunctional HDL was associated with active disease status and altered HDL protein cargo in RA [12]. Arts et al. also demonstrated that changed HDL composition through the effect of inflammation on HDL subfractions may contribute to a high ASCVD risk in RA patients [13]. Besides, recent advances in lipid/metabolomics facilitate the characterization of lipoprotein subfractions and quantification of lipid metabolites in blood samples or vascular plaques [14,15]. Therefore, a lipid/metabolomics signature would be a promising biomarker for ASCVD and serve as a new therapeutic target [16]. However, the changes in HDL size and HDL-related metabolites in RA patients have yet to be fully studied using lipid/metabolomics. Besides, given that anti-citrullinated peptide antibody (ACPA) positive RA patients exhibit a higher risk of ASCVD compared to those without ACPA [17,18], lipid/metabolomics might be a useful tool to find the differential HDL-related metabolites linked to ACPA positivity.

In this pilot study, we aimed to use ^1^H-NMR-based lipid/metabolomics to identify the significantly differential HDL-related metabolites between RA patients and healthy control (HC) subjects. We also evaluated the association between HDL-related metabolite levels and the RA inflammatory index and the impact of Janus kinase inhibitor (JAKi) therapy on these metabolites.

## 2. Results

### 2.1. Clinical Characteristics of RA Patients and HC Participants

As illustrated in Table 1, there were no significant differences in age at study entry, the proportion of females, body mass index, or lipid profile between RA patients and HC participants. There was also no significant difference in the proportion of statin use, comorbidities, or current smoking status between RA patients and HC participants.

Besides, we dichotomized RA patients based on ACPA positivity and compared the differences in demographics, disease activity, and lipid profiles between ACPA-positive and ACPA-negative patients. As illustrated in Supplementary Table S2, a significantly higher degree of inflammation, ESR, and lower levels of total cholesterol and triglycerides were observed in ACPA-positive patients than in ACPA-negative patients. There were no significant differences in age at study entry, the proportion of females, body mass index, the proportion of comorbidities, or current smoking status between ACPA-positive and ACPA-negative patients.

### 2.2. The Discriminant HDL-Related Lipid Metabolites between RA Patients and HC Subjects

**Figure 1:**
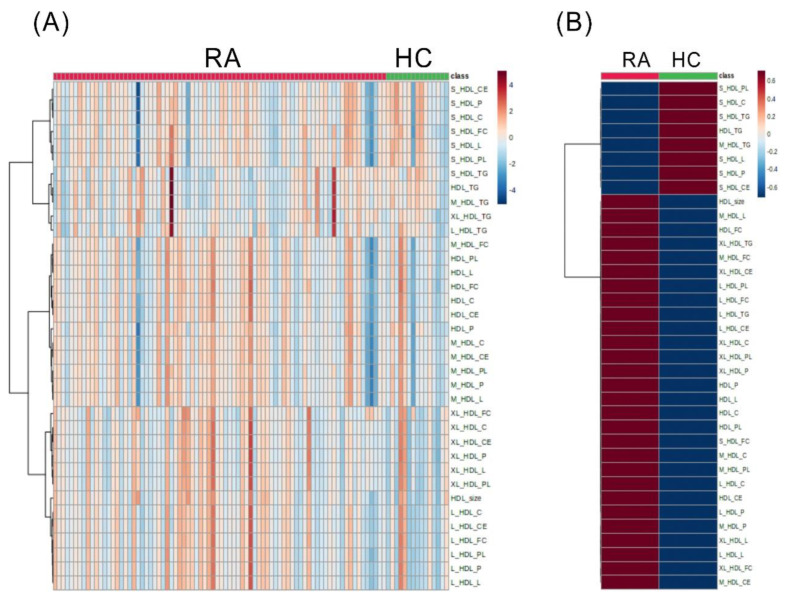
The differences in the levels of discriminant HDL-related metabolites between RA and HC subjects. Classification of subjects according to lipidomic data heatmap representing relative amounts (**A**) and the mean levels (**B**) of discriminant HDL-related metabolites between rheumatoid arthritis (RA) and healthy control (HC) subjects. Each metabolite’s level is color-coded on a normalized scale ranging from a minimum of −4 (dark blue) to a maximum of 4 (dark red). Rows indicate HDL-related metabolites and columns indicate subjects.

In the present study, the disparities in HDL-related metabolites were evaluated between RA and HC subjects. A total of 36 HDL-related metabolites were analyzed, and a list of HDL-related markers is illustrated in Supplementary Table S1. Based on hierarchical cluster analysis of the 36 HDL-related metabolites in RA and HC subjects, the heatmap displays the relative amounts of discriminant metabolites (Figure 1A) and the mean levels (Figure 1B). Compared with HC subjects, RA patients exhibited a higher level of particle number (P), total lipids (L), cholesterol (C), cholesterol ester (CE), free cholesterol (FC), and phospholipids (PL) in large- or very large-sized HDLs. The detailed differences between RA patients and HC subjects are presented in Supplementary Figure S1.

### 2.3. Comparison of the Levels of HDL-Related Metabolites between RA Patients with and without ACPA or between Patients with and without RF

**Figure 2:**
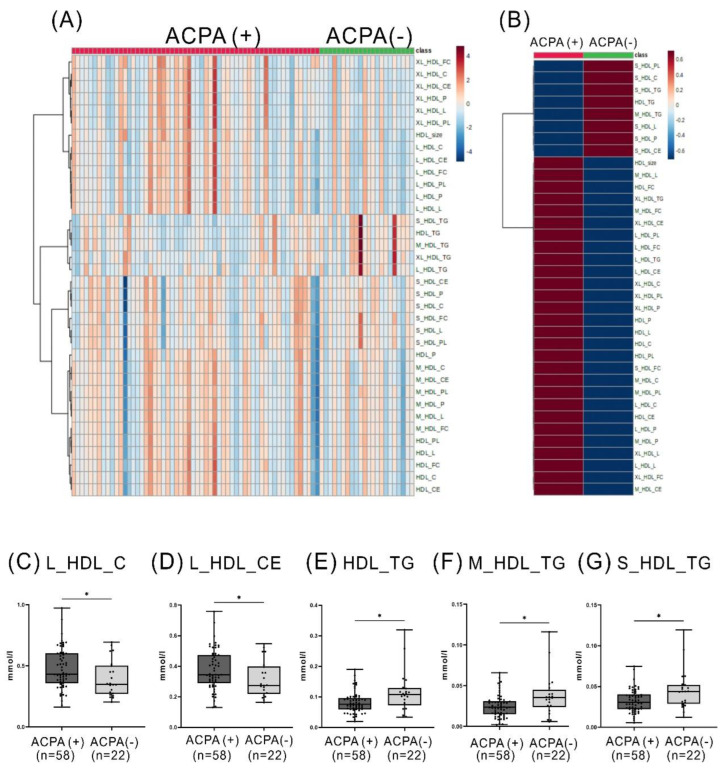
The difference in the levels of discriminant HDL-related metabolites between RA patients with and without ACPA. Classification of subjects according to lipidomic data heatmap representing relative amounts (**A**) and the mean levels (**B**) of discriminant HDL-related metabolites between ACPA-positive and ACPA-negative rheumatoid arthritis (RA) patients. Each metabolite’s concentration is color-coded on a normalized scale ranging from a minimum of −4 (dark blue) to a maximum of 4 (dark red). The difference in significantly expressed metabolites between patients with and without ACPA (**C**–**G**). * *p* < 0.05, determined by using Mann–Whitney U test.

Given that ACPA-positive RA patients exhibit a higher risk of atherosclerosis compared to those without ACPA [17,18], we examined the association between HDL-related metabolites and the positivity of ACPA. The lipidomic data heatmap illustrates the relative amounts (Figure 2A) and the mean levels of discriminant HDL-related metabolites between ACPA-positive and ACPA-negative RA patients. Besides, we compared the difference in significantly expressed metabolites between patients with and without ACPA (Figure 2C–G). RA patients with positive ACPA had significantly higher levels of large-sized HDL-C and large-sized HDL-CE but lower levels of small- or medium-sized HDL-TG than those without ACPA.

We also dichotomized RA patients based on RF positivity and compared the difference in HDL-related metabolites. The results showed no significant difference in serum levels of large-sized HDL-C, large-sized HDL-CE, or small-/medium-sized HDL-TG between RF-positive and RF-negative patients, as shown in Supplementary Figure S2.

### 2.4. The Relationship among the Discriminant HDL-Related Metabolites in RA Patients

**Figure 3:**
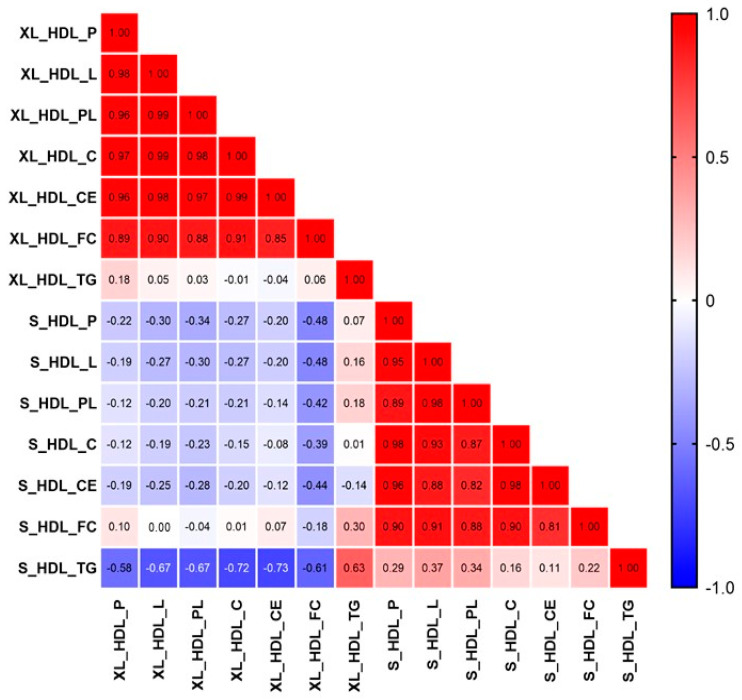
The relationship among the discriminant HDL-related metabolites in RA patients.

To evaluate the relationship among distinct metabolites, we conducted a correlation analysis on the HDL-associated metabolites in RA patients. As shown in Figure 3, a negative correlation was observed between small-sized and very large-sized HDL-related metabolites.

### 2.5. The Correlation between Inflammatory Parameter, Represented by CRP Levels, and HDL-Related Metabolites in RA Patients

As illustrated in Table 2, a negative correlation was observed between serum CRP levels and small/medium-sized HDL-related metabolites, the athero-protective metabolites in RA patients. CRP levels were also inversely correlated with TG in medium-sized, large-sized, and very large-sized HDL.

Besides, we re-evaluated the correlation between CRP levels and HDL-related metabolites in 66 patients without using statins at baseline. The results still revealed an inverse correlation between serum CRP levels and small/medium-sized HDL-related metabolites, the athero-protective metabolites in RA patients, as illustrated in Supplementary Table S3.

### 2.6. Changes of HDL-Related Metabolites in RA Patients Treated with JAKi

**Figure 4:**
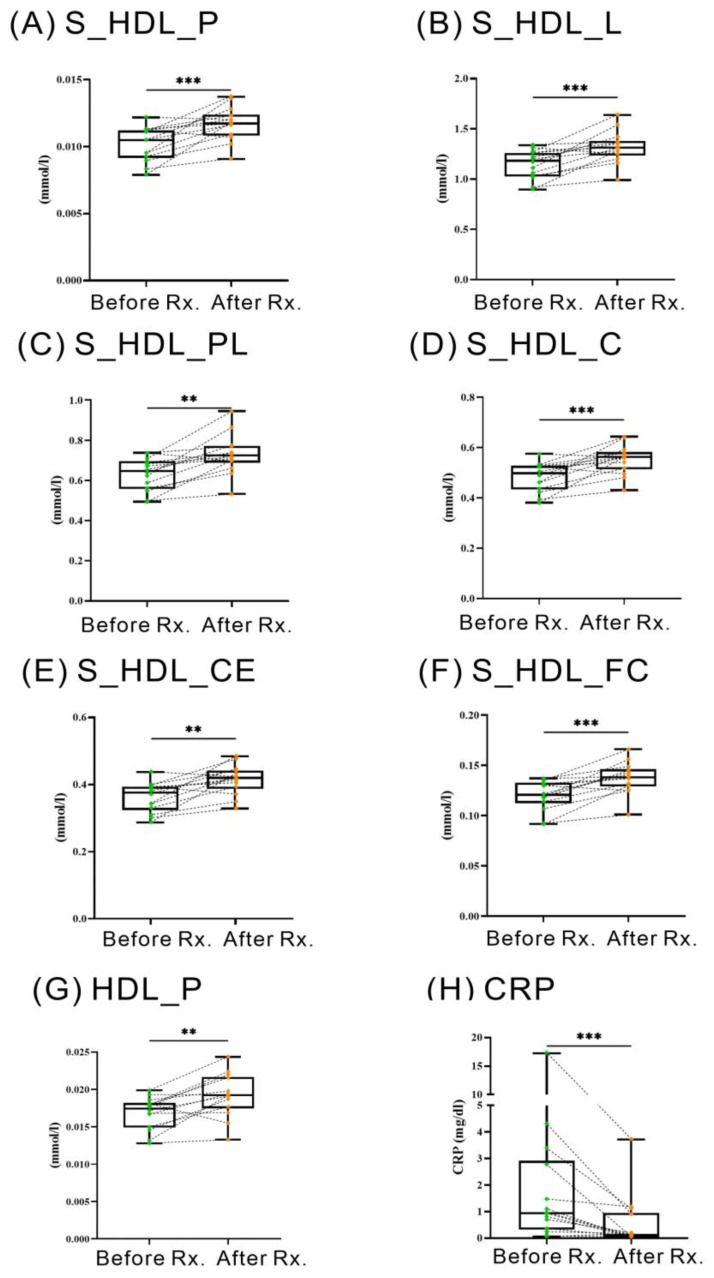
The change in HDL metabolites and CRP level after 6–12 months of Janus kinase inhibitor therapy in rheumatoid arthritis patients (*n* = 14). ** *p* < 0.01, *** *p* < 0.005, determined by using Wilcoxon matched-pairs signed-rank test.

Fourteen patients were available for examination of HDL-related metabolites before and after 6–12 months of therapy with Janus kinase inhibitors (JAKi), including twelve patients receiving tofacitinib and two receiving baricitinib. Figure 4 illustrates that the treatment with JAKi resulted in a significant augmentation in serum levels of total HDL particle number (HDL_P) and small-sized HDL particle number (S_HDL_P) and small-sized HDL-related metabolites, including total lipids, phospholipids, cholesterol, cholesterol ester, and free cholesterol (S_HDL_L, S_HDL_PL, S_HDL_C, S_HDL_CE, and S_HDL_FC). This enhancement was concomitant with a reduction in disease activity, as indicated by CRP levels (median 0.95 mg/dL, interquartile range (IQR) 0.33–2.92 mg/dL versus 0.14 mg/dL, IQR 0.05–0.96 mg/dL, *p* <0.005).

Among the fourteen patients treated with JAKi, nine received combined therapy with csDMARDs, and five received monotherapy. As illustrated in Supplementary Table S4, HDL-related metabolite changes from baseline in JAKi combined therapy were similar to the changes in fourteen JAKi-treated patients. Despite a lack of statistical significance, JAKi monotherapy was also associated with increased serum levels of total HDL particle number (HDL_P), small-sized HDL particle number (S_HDL_P), and small-sized HDL-related metabolites, including total lipids, phospholipids, cholesterol, cholesterol ester, and free cholesterol (S_HDL_L, S_HDL_PL, S_HDL_C, S_HDL_CE, and S_HDL_FC), as illustrated in Supplementary Table S4.

Although a total of forty patients received biologics therapy (TNF inhibitors, tocilizumab, abatacept, and rituximab were used in seventeen, sixteen, six, and one patient, respectively), only five (three TNFi- and two tocilizumab-treated patients) were available for examination in HDL-related metabolites before and after 6–12 months of biologics therapy. Given the small sample size, we did not analyze the changes in HDL-related metabolites in RA patients treated with biologics.

## 3. Discussion

Despite the common perception that HDL particles are athero-protective [2,3], increasing evidence indicates that such protective functions vary with the different sizes or composition of HDL-c [5,6]. Chronic inflammation may also cause changes in the HDL structure and affect its athero-protective functions [6]. Herein, we demonstrated for the first time that the sizes and composition of HDL-related metabolites were significantly different between RA patients and HC subjects. The hierarchical clustering plot revealed that RA patients exhibited a higher proportion of large- or very large-sized HDLs than HC subjects. Furthermore, ACPA-positive RA patients had significantly higher levels of large-sized HDL-C and large-sized HDL-CE compared with ACPA-negative patients. The correlation analysis showed an inverse correlation between the degree of inflammation, as reflected by CRP levels, and the levels of athero-protective small-sized HDLs. The athero-protective small-sized HDL-related metabolites were also increased after effective treatment with JAKi. These observations suggest a significant association between inflammation and the size and composition of HDL-related metabolites in RA patients.

RA, a chronic inflammatory arthritis, is associated with a 50–70% higher risk of ASCVD than healthy individuals [7,19]. Using ^1^H-NMR-based lipidomics, we revealed a dominance of large-sized or very large-sized HDL-related metabolites in RA patients but not in HC subjects. RA patients also have significantly higher levels of HDL-related metabolites, including cholesterol, CE, total lipids, FC, and PL, in large-sized HDL particles than HC subjects. With increased activity of phospholipid transfer protein (PLTP) in RA patients [20], this protein would cause PL accumulation in HDL particles and hence enlargement of their sizes [21,22]. Accordingly, our RA patients had a higher proportion of large-sized HDL particles and higher levels of HDL-related PL than HC subjects. Ferraz-Amaro et al. reported reduced activity of cholesteryl ester transfer protein (CETP) in RA patients [23], which could lead to CE accumulation in HDL particles [24,25]. We similarly found higher CE levels in large-sized HDL particles in RA patients than in HC subjects. Besides, impaired CETP function may impede FC-to-CE conversion in HDL [26], as revealed in our results showing a higher FC level in large-sized HDL particles in RA patients than in HC subjects.

The link between ACPA positivity and HDL-related metabolites has yet to be fully explored. Compared with ACPA-negative RA patients, our ACPA-positive patients had significantly higher levels of cholesterol (C) and CE in large-sized HDL particles, so-called dysfunctional HDL [25,27,28]. A significantly lower level of HDL-associated TG was also observed in ACPA-positive RA patients than in our ACPA-negative patients, resonating with the findings of McLaren et al. that reduced CETP activity may impede the transfer of TG from LDL/VLDL to HDL and cause decreased TG content within HDL particles [29]. CETP also has anti-inflammatory properties and may be depleted or reduced in inflammatory conditions [8,30]. Besides, APCA positivity is linked to ASCVD risk in RA patients [17,18]. Higher degrees of inflammation and ESR observed in our ACPA-positive patients than in ACPA-negative patients may contribute to ASCVD risk since high ESR is associated with increased ASCVD risk [31]. These observations suggest that ACPA-positive RA patients may exhibit elevated levels of dysfunctional HDL, a highly inflammatory environment, and increased ASCVD risk compared to ACPA-negative patients. These observations suggest that ACPA-positive RA patients may exhibit a more severe inflammatory environment compared to ACPA-negative patients. Nevertheless, the exact mechanism for the link between ACPA positivity and HDL-related metabolites in RA patients awaits further investigation.

Chronic inflammation may modify the structure and athero-protective functions of HDL [6]. Dysfunctional HDL was associated with an active disease status of RA [12], and HDL composition could change under the influence of inflammation in RA patients [13]. In the present study, we demonstrated an inverse correlation between CRP levels and athero-protective small-/medium-sized HDL-related metabolites in RA patients, resonating with previous reports of a negative correlation between HDL concentrations, particularly small-size HDL, and CRP levels in RA patients [32,33,34,35]. These observations suggest an association between inflammatory status and the sizes and composition of HDL-related metabolites in RA patients.

The JAKi, including tofacitinib and baricitinib, are effective in RA treatment and may markedly reduce CRP levels [36,37,38]. Charles-Schoeman et al. revealed that RA patients treated with JAKi experienced a significant reduction in the inflammatory marker CRP, an elevation of HDL levels, and a concurrent improvement in HDL function [39]. We likewise demonstrated increased HDL levels and a proportion of small-sized HDL in RA patients receiving JAKI treatment, which may reflect the restoration of HDL function. Our results also resonate with previous findings that tofacitinib therapy could increase HDL particle number [39] and that baricitinib therapy augmented small-sized HDL [40].

Despite the novel findings in this pilot study, there are still some limitations. First, all subjects in this study are of Chinese ethnicity, and our findings may not be generalizable to other ethnic groups. Given a small sample size of ACPA-negative patients, we did not perform multivariate analysis. The treatment with concomitant corticosteroids, csDMARDs, or statins may affect lipid metabolites [41,42,43], and we did not evaluate the relationship between lipid metabolite levels and the therapy with these medications. Given the small sample size of RA patients, in whom the rate of CVD emergence observed was low, we could not evaluate the relationship between the distinct profiling of HDL-related metabolites and ASCVD risk in this disease. Therefore, a future long-term, large-scale study is required to confirm our findings.

## 4. Materials and Methods

### 4.1. Subjects

This investigation included eighty RA participants, each of whom met the updated criteria set forth by the American College of Rheumatology in 2010 for the diagnosis of RA [44]. The determination of disease activity was achieved by the application of the 28-joint disease activity score (DAS28) [45], and active disease was subsequently characterized by a DAS28-erythrocyte sedimentation rate (DAS28-ESR) score that equals or surpasses a threshold of 3.2. After baseline lipid/metabolomics investigation, seventy-six active RA patients who had a poor therapeutic response to conventional synthetic DMARDs (csDMARDs) started biologic DMARD (bDMARDs) or Janus kinase inhibitor (JAKi) therapy according to the recommendations [46], and the other four patients continued with csDMARD treatment alone. The study included fifteen healthy controls (HC), selected based on matching age and sex and with no history of rheumatic disease. This investigation received the necessary endorsement from the Research Ethics Center at China Medical University and Hospital (CMUH109-REC3-161, approval date 13 December 2020). In accordance with the ethical guidelines stipulated by the Declaration of Helsinki, written consent was procured from every individual participant.

### 4.2. Lipid Profile Assessment and Atherogenic Index Calculation

Blood specimens from participants were collected in the early morning hours, adhering to the requirement of a 12 h fasting period preceding collection. Subsequent evaluation of plasma levels encompassed the measurement of total cholesterol (TC), triglycerides (TG), high-density lipoprotein cholesterol (HDL-c), and low-density lipoprotein cholesterol (LDL-c). These evaluations were accurately carried out utilizing enzymatic methodologies on a Beckman Coulter AU5800 chemistry analyzer (Brea, CA, USA), strictly following the guidance provided by the manufacturer’s instructions. Thereafter, an assessment of the atherogenic index was performed by precisely calculating the ratio of TC to HDL-c.

### 4.3. Employment of ^1^H-NMR Lipid/Metabolomics for the Determination of Serum Lipid Metabolites

Serum specimens were subjected to a comprehensive analysis employing ^1^H-NMR lipid/metabolomics (Nightingale Health, Helsinki, Finland), following a procedure similar to previous studies [16]. Initial steps involved mixing the samples with phosphate buffer, after which they were carefully transferred to a 3 mm NMR tube. Measurements were subsequently undertaken at a temperature of 310 K using the highly sophisticated Bruker Avance III NMR spectrometers, operating at a frequency of 600.13 MHz and equipped with a maximum gradient strength of 53 G/cm. Following a vital equilibration period of 5 min, automatic processing of the data sets was undertaken using a line broadening of 1 Hz, and alignment of the nuclear Overhauser enhancement spectroscopy data was achieved with reference to the alanine signal resonating at 1.49 ppm.

### 4.4. Data Processing

The NMR data were removed from the ratio in the lipoprotein subgroup to avoid errors caused by repeated calculations, as described in the previous literature [47]. A heatmap representation based on hierarchical clustering analysis was meticulously constructed with the aid of MetaboAnalyst 5.0 (https://www.metaboanalyst.ca/ (accessed on 1 April 2023)), a web-based analytical tool. The process involved the utilization of Euclidean distance measures coupled with Ward’s minimum variance method to ensure rigorous data clustering.

### 4.5. Statistical Analysis

The data were presented as the mean ± standard deviation (SD) or the median (interquartile range, IQR). The Mann–Whitney U test was applied to evaluate statistical variations in lipid profiles, demographic information, laboratory data, and HDL-related metabolites specifically among the two distinct groups being investigated. Derivation of the correlation coefficient was achieved by applying the nonparametric Spearman’s rank correlation test, which is recognized for its robustness in assessing associations. Subsequently, the Wilcoxon matched-pairs signed-rank test, a non-parametric statistical hypothesis test, was used to evaluate the alterations in levels of HDL-related metabolites among RA patients post JAKi treatment. For all statistical evaluations carried out in this study, a two-sided *p*-value of less than 0.05 was regarded as indicative of statistical significance. Furthermore, the construction of data visualizations and execution of statistical analyses were accomplished through the software tools, specifically IBM SPSS Statistics v25 (IBM, New York, NY, USA) and GraphPad Prism v9.5 (GraphPad Software, San Diego, CA, USA).

## 5. Conclusions

This study is the first to reveal that the sizes of HDL particles and the composition of HDL-related metabolites are significantly associated with the inflammatory status of RA. A potential link was observed between ACPA positivity and changes in HDL-related metabolites in RA patients. JAKi treatment was associated with increased HDL particle number and serum levels of small-sized HDL metabolites. Nevertheless, the causative role of inflammation in the size or composition changes of HDL particles in RA still needs further investigation.

## Figures and Tables

**Table 1:** Demographic and laboratory data in rheumatoid arthritis (RA) patients, healthy controls (HC) ^a^.

	RA (*n* = 80)	HC (*n* = 15)	*p*-Value
Age at entry, years	56.1 ± 9.5	48.5 ± 13.1	0.0525
Gender (female), n (%)	64 (80.0%)	10 (66.7%)	0.3098
Body mass index, kg/m^2^	23.8 ± 4.0	23.0 ± 2.2	0.6646
Disease duration (months)	75.0 ± 29.3	NA	
RF positive, n (%)	60 (75.0%)	NA	
ACPA positive, n (%)	58 (72.5%)	NA	
ESR, mm/h	23.5 (13.0–35.5)	NA	
C reactive protein, mg/dL	0.83 (0.12–1.81)	NA	
TC, mg/dL	201.0 (168.0–228.0)	182.0 (148.0–225.0)	0.4466
TG, mg/dL	89.5 (64.3–131.8)	81.5 (42.5–147.5)	0.4469
HDL-C, mg/dL	61.5 (49.9–72.9)	51.3 (44.5–75.5)	0.4438
LDL-C, mg/dL	120.2 (87.6–143.4)	121.7 (90.4–151.4)	0.9279
Atherogenic index	3.23 (2.62–4.25)	3.69 (2.59–4.20)	0.6999
Stains treatment	14 (17.5%)	1 (6.7%)	0.4519
Concomitant csDMARDs			
Methotrexate, n (%)	64 (80.0%)	NA	
Hydroxychloroquine, n (%)	51 (63.8%)	NA	
Sulfasalazine, n (%)	48 (60.0%)	NA	
The use of bDMARDs			
TNFi plus csDMARDs, n (%)	17 (21.3%)	NA	
Tocilizumab plus csDMARDs, n (%)	11 (13.8%)	NA	
Tocilizumab monotherapy, n (%)	5 (6.3%)	NA	
Abatacept plus csDMARDs, n (%)	6 (7.5%)	NA	
Rituximab plus csDMARDs, n (%)	1 (1.3%)	NA	
The use of tsDMARDs			
Tofacitinib plus csDMARDs, n (%)	26 (32.5%)	NA	
Tofacitinib monotherapy, n (%)	4 (5.0%)	NA	
Baricitinib plus csDMARDs, n (%)	3 (3.8%)	NA	
Baricitinib monotherapy, n (%)	1 (1.3%)	NA	
Upatacitinib plus csDMARDs, n (%)	2 (2.5%)	NA	
Comorbidities, n (%)			
Hypertension, n (%)	15 (18.8%)	1 (6.7%)	0.4532
Diabetes mellitus, n (%)	3 (3.8%)	0 (0%)	>0.9999
Current smoker, n (%)	11 (13.8%)	1 (6.7%)	0.6837

^a^ Data are presented as mean ± SD, median (interquartile range, IQR), number (%); RF: rheumatoid factor; ACPA: anti-citrullinated peptide antibodies; ESR: erythrocyte sedimentation rate; TC: total cholesterol; HDL-C: high-density lipoprotein cholesterol; TG: triglyceride; LDL-C: low-density lipoprotein cholesterol; atherogenic index: the ratio of TC /HDL-C; csDMARDs: conventional synthetic disease-modifying anti-rheumatic drugs; tsDMARDs: targeted synthetic DMARDs; TNFi: tumor necrosis factor inhibitors; NA: Not applicable.

**Table 2:** The correlation between serum levels of C-reactive protein (CRP) and HDL-related metabolites in RA patients.

	CRP Levels			CRP Levels	
Small-Sized HDL Composition	r Value	*p*-Value	Medium-Sized HDL Composition	r Value	*p*-Value
Particle number (P)	**−0.411**	**<0.001**	Particle number (P)	**−0.225**	**0.045**
Total lipids (L)	**−0.38**	**<0.001**	Total lipids (L)	−0.206	0.067
Phospholipids (PL)	**−0.338**	**0.002**	Phospholipids (PL)	−0.190	0.091
Cholesterol (C)	**−0.403**	**<0.001**	Cholesterol (C)	−0.191	0.090
Cholesteryl ester (CE)	**−0.382**	**<0.001**	Cholesteryl ester (CE)	−0.183	0.104
Free cholesterol (FC)	**−0.433**	**<0.001**	Free cholesterol (FC)	**−0.225**	**0.045**
Triglycerides (TG)	−0.163	0.150	Triglycerides (TG)	**−0.254**	**0.023**
	**CRP Levels**			**CRP Levels**	
**Large-Sized HDL Composition**	**r Value**	***p*-Value**	**Very Large-Sized HDL Composition**	**r Value**	***p*-Value**
Particle number (P)	−0.110	0.333	Particle number (P)	−0.116	0.305
Total lipids (L)	−0.032	0.776	Total lipids (L)	−0.095	0.404
Phospholipids (PL)	0.006	0.960	Phospholipids (PL)	−0.078	0.490
Cholesterol (C)	−0.046	0.684	Cholesterol (C)	−0.087	0.443
Cholesteryl ester (CE)	−0.073	0.519	Cholesteryl ester (CE)	−0.088	0.438
Free cholesterol (FC)	0.016	0.888	Free cholesterol (FC)	−0.088	0.438
Triglycerides (TG)	**−0.223**	**0.047**	Triglycerides (TG)	**−0.277**	**0.013**

The correlation analysis of CRP and HDL-related metabolites of different sizes of HDL. Statistically significant differences are marked in bold (*p* < 0.05), and the statistical method is Spearman correlation analysis.

## Data Availability

The data supporting this study are available in the article, the supplemental data, or from the corresponding author upon reasonable request.

## Supplementary Materials

The following supporting information can be downloaded at: https://www.mdpi.com/article/10.3390/ijms241310645/s1.

## Abbreviations

ACPA	Anti-citrullinated peptide antibodies
ASCVD	Atherosclerotic cardiovascular diseases
bDMARDs	Biologic DMARDs
C	Cholesterol
CE	Cholesterol ester
CETP	Cholesteryl ester transfer protein
CRP	C-reactive protein
csDMARDs	Conventional synthetic DMARDs
DAS28	28-joint disease activity score
FC	Free cholesterol
HC	Healthy control
HDL	High-density lipoprotein
HDL-c	High-density lipoprotein cholesterol
IQR	Interquartile range
JAKi	Janus kinase inhibitors
L	Total lipids
LC/MS	Liquid chromatography/mass spectrometry
LDL-c	Low-density lipoprotein cholesterol
L-HDL	Large-sized HDLs
P	Particle number
PL	Phospholipids
PLTP	Phospholipid transfer protein
RA	Rheumatoid arthritis
RF	Rheumatoid factor
XL-HDL	Very large-sized HDLs
