# Supplementary material for: The Sizes and Composition of HDL-Cholesterol Are Significantly Associated with Inflammation in Rheumatoid Arthritis Patients
Source: Int J Mol Sci. 2023 Jun 26;24(13):10645. doi: 10.3390/ijms241310645 (PMC10341560; doi:10.3390/ijms241310645)
Supplement: Supplementary file 1 [file ijms-24-10645-s001.zip › ijms-2451230-supplementary.pdf]

Table S1. The list of marks in HDL-related lipid metabolites

Short name	Descriptions	Unit
HDL_C	HDL cholesterol	mmol/l
HDL_TG	Triglycerides in HDL	mmol/l
HDL_PL	Phospholipids in HDL	mmol/l
HDL_CE	Cholesteryl esters in HDL	mmol/l
HDL_FC	Free cholesterol in HDL	mmol/l
HDL_L	Total lipids in HDL	mmol/l
HDL_P	Concentration of HDL particles	mmol/l
HDL size	Average diameter for HDL particles	nm
XL_HDL_P	Concentration of very large HDL particles	mmol/l
XL_HDL_L	Total lipids in very large HDL	mmol/l
XL_HDL_PL	Phospholipids in very large HDL	mmol/l
XL_HDL_C	Cholesterol in very large HDL	mmol/l
XL_HDL_CE	Cholesteryl esters in very large HDL	mmol/l
XL_HDL_FC	Free cholesterol in very large HDL	mmol/l
XL_HDL_TG	Triglycerides in very large HDL	mmol/l
L_HDL_P	Concentration of large HDL particles	mmol/l
L_HDL_L	Total lipids in large HDL	mmol/l
L_HDL_PL	Phospholipids in large HDL	mmol/l
L_HDL_C	Cholesterol in large HDL	mmol/l
L_HDL_CE	Cholesteryl esters in large HDL	mmol/l
L_HDL_FC	Free cholesterol in large HDL	mmol/l
L_HDL_TG	Triglycerides in large HDL	mmol/l
M_HDL_P	Concentration of medium HDL particles	mmol/l
M_HDL_L	Total lipids in medium HDL	mmol/l
M_HDL_PL	Phospholipids in medium HDL	mmol/l
M_HDL_C	Cholesterol in medium HDL	mmol/l
M_HDL_CE	Cholesteryl esters in medium HDL	mmol/l
M_HDL_FC	Free cholesterol in medium HDL	mmol/l
M_HDL_TG	Triglycerides in medium HDL	mmol/l
S_HDL_P	Concentration of small HDL particles	mmol/l
S_HDL_L	Total lipids in small HDL	mmol/l
S_HDL_PL	Phospholipids in small HDL	mmol/l
S_HDL_C	Cholesterol in small HDL	mmol/l
S_HDL_CE	Cholesteryl esters in small HDL	mmol/l

S_HDL_FC	Free cholesterol in small HDL	mmol/l
S_HDL_TG	Triglycerides in small HDL	mmol/l

Table S2. Baseline demographic and laboratory data in rheumatoid arthritis (RA) patients with positive anti-citrullinated peptide antibodies (ACPA) and negative ACPA.

	**ACPA (+) (n=58)**	**ACPA (-) (n=22)**	***p* value**
Age at entry, years	56.2 ± 9.4	56.0 ± 10.2	0.9808
Gender (female), n (%)	46 (79.3%)	18 (81.8%)	>0.9999
Body mass index, kg/m^2	23.5 ± 4.3	24.4 ± 3.1	0.2003
Disease duration (months)	73.2 ± 26.1	79.9 ± 36.6	0.7020
**ESR, mm/hr**	**25.0 (16.0-37.3)**	**13.5 (10.3-25.8)**	**0.0137**
C-reactive protein, mg/dl	1.03 (0.16-1.88)	0.54 (0.04-1.19)	0.1854
DAS28 score at baseline	6.14 (5.44-6.73)	5.68 (3.80-6.76)	0.2454
**Total cholesterol, mg/dl**	**194.0 (163.5-227.5)**	**217.0 (193.0-239.0)**	**0.0498**
**Triglyceride, mg/dl**	**80.0 (54.5-111.0)**	**121.0 (88.5-157.5)**	**0.0005**
HDL-C, mg/dl	61.5 (50.8-72.4)	62.5 (48.4-75.2)	0.8789
LDL-C, mg/dl	116.2 (84.4-142.6)	129.4 (102.8-146.3)	0.3149
Atherogenic index	3.12 (2.53-4.16)	3.33 (2.90-4.67)	0.1463
Comorbidities, n (%)			
Hypertension, n (%)	10 (17.2%)	5 (22.7%)	0.7490
Diabetes mellitus, n (%)	3 (5.2%)	0 (0%)	0.5574
Current smoker, n (%)	9 (15.5%)	2 (9.1%)	0.7183

[a] Data are presented as mean ± SD, median (interquartile range, IQR), number (%); ACPA: anti-citrullinated peptide antibodies; ESR: erythrocyte sedimentation rate; HDL-C: high-density lipoprotein cholesterol; LDL-C: low-density lipoprotein cholesterol; Atherogenic index: the ratio of TC/HDL-C.

Table S3. The correlation between serum levels of C-reactive protein (CRP) and HDL-related metabolites in RA patients without use of statins at baseline (n=66).

	**CRP levels**			**CRP levels**	
**Small-size HDL composition**	r value	*p* value	**Medium-size HDL composition**	r value	*p* value
Particle number (P)	**-0.422**	**<0.001**	Particle number (P)	**-0.332**	**0.007**
Total lipids (L)	**-0.399**	**<0.001**	Total lipids (L)	**-0.297**	**0.016**
Phospholipids (PL)	**-0.373**	**<0.001**	Phospholipids (PL)	**-0.275**	**0.025**
Cholesterol (C)	**-0.430**	**<0.001**	Cholesterol (C)	**-0.300**	**0.014**
Cholesteryl ester (CE)	**-0.397**	**<0.001**	Cholesteryl ester (CE)	**-0.288**	**0.019**
Free cholesterol (FC)	**-0.467**	**<0.001**	Free cholesterol (FC)	**-0.335**	**0.006**
Triglycerides (TG)	-0.094	0.454	Triglycerides (TG)	-0.214	0.084
	**CRP levels**			**CRP levels**	
**Large-size HDL composition**	r value	*p* value	**Very large-size HDL composition**	r value	*p* value
Particle number (P)	**-0.246**	**0.047**	Particle number (P)	-0.192	0.122
Total lipids (L)	-0.216	0.082	Total lipids (L)	-0.113	0.366
Phospholipids (PL)	-0.198	0.111	Phospholipids (PL)	-0.076	0.545
Cholesterol (C)	-0.218	0.079	Cholesterol (C)	-0.123	0.324
Cholesteryl ester (CE)	-0.217	0.080	Cholesteryl ester (CE)	-0.165	0.187
Free cholesterol (FC)	-0.203	0.101	Free cholesterol (FC)	-0.025	0.843
Triglycerides (TG)	-0.303	0.013	Triglycerides (TG)	-0.230	0.064

The correlation analysis of CRP and HDL-related metabolites of different sizes of HDL. Statistically significant differences are marked in bold ($p<0.05$), and the statistical method is Spearman correlation analysis.

Table S4. The change of HDL-related metabolites and CRP levels after 6-12 months of Janus kinase inhibitors (JAKi) combined with csDMARDs therapy or monotherapy in rheumatoid arthritis patients.

	**JAKi combined with csDMARDs (n=9)**			**JAKi monotherapy (n=5)**		
	Baseline	After therapy	p-Value	Baseline	After therapy	p-Value
HDL_C	1.543 (1.458-1.958)	1.759 (1.642-2.083)	0.098	1.773 (1.443-1.790)	1.829 (1.750-1.872)	0.438
HDL_TG	0.081 (0.052-0.099)	0.112 (0.051-0.129)	0.129	0.072 (0.071-0.116)	0.113 (0.052-0.129)	0.438
HDL_PL	1.532 (1.491-1.892)	1.772 (1.700-2.145)	0.074	1.771 (1.497-1.811)	1.869 (1.820-1.870)	0.125
HDL_CE	1.228 (1.142-1.537)	1.376 (1.304-1.663)	0.098	1.410 (1.142-1.415)	1.434 (1.383-1.499)	0.625
HDL_FC	0.317 (0.310-0.413)	0.356 (0.332-0.420)	0.129	0.358 (0.301-0.369)	0.373 (0.367-0.396)	0.125
HDL_L	3.155 (3.061-3.885)	3.655 (3.464-4.340)	0.074	3.615 (3.057-3.719)	3.743 (3.732-3.852)	0.125
**HDL_P**	**0.017 (0.015-0.018)**	**0.019 (0.018-0.022)**	**0.024**	0.018 (0.017-0.019)	0.019 (0.019-0.020)	0.100
HDL_size	9.809 (9.694-9.901)	9.722 (9.590-9.848)	0.055	9.698 (9.675-9.791)	9.764 (9.697-9.828)	0.625
XL_HDL_P	0.000 (0.000-0.000)	0.000 (0.000-0.000)	0.055	0.000 (0.000-0.000)	0.000 (0.000-0.000)	0.313
**XL_HDL_L**	**0.202 (0.175-0.245)**	**0.168 (0.129-0.194)**	**0.039**	0.176 (0.160-0.214)	0.213 (0.169-0.219)	0.498
**XL_HDL_PL**	**0.098 (0.080-0.119)**	**0.074 (0.048-0.094)**	**0.020**	0.080 (0.068-0.102)	0.095 (0.075-0.103)	0.625
**XL_HDL_C**	**0.101 (0.091-0.120)**	**0.091 (0.073-0.093)**	**0.039**	0.091 (0.089-0.108)	0.107 (0.088-0.112)	0.813
XL_HDL_CE	0.076 (0.070-0.097)	0.074 (0.057-0.078)	0.098	0.070 (0.069-0.085)	0.085 (0.068-0.090)	0.813
**XL_HDL_FC**	**0.025 (0.023-0.028)**	**0.018 (0.014-0.021)**	**0.004**	0.021 (0.020-0.024)	0.022 (0.021-0.022)	0.892
XL_HDL_TG	0.006 (0.005-0.006)	0.005 (0.004-0.007)	0.910	0.005 (0.003-0.007)	0.005 (0.005-0.007)	0.313

L_HDL_P	0.002 (0.002-0.003)	0.002 (0.002-0.003)	0.910	0.002 (0.002-0.002)	0.002 (0.002-0.002)	0.361
L_HDL_L	0.811 (0.760-1.226)	0.921 (0.706-1.068)	0.820	0.851 (0.702-0.988)	0.990 (0.872-1.061)	0.313
L_HDL_PL	0.390 (0.345-0.586)	0.438 (0.335-0.507)	0.734	0.408 (0.318-0.466)	0.464 (0.426-0.499)	0.438
L_HDL_C	0.401 (0.388-0.622)	0.479 (0.350-0.537)	0.910	0.430 (0.371-0.497)	0.492 (0.422-0.553)	0.813
L_HDL_CE	0.320 (0.310-0.496)	0.388 (0.282-0.423)	>0.999	0.344 (0.300-0.393)	0.390 (0.334-0.443)	0.813
L_HDL_FC	0.080 (0.078-0.126)	0.092 (0.068-0.114)	0.734	0.086 (0.071-0.103)	0.103 (0.088-0.110)	0.361
L_HDL_TG	0.018 (0.011-0.021)	0.022 (0.012-0.025)	>0.999	0.013 (0.012-0.019)	0.021 (0.018-0.024)	0.188
M_HDL_P	0.004 (0.004-0.005)	0.005 (0.005-0.006)	0.074	0.005 (0.004-0.005)	0.005 (0.005-0.005)	0.125
**M_HDL_L**	**1.040 (0.958-1.258)**	**1.252 (1.161-1.560)**	**0.039**	1.246 (1.046-1.263)	1.294 (1.260-1.318)	0.125
**M_HDL_PL**	**0.464 (0.428-0.568)**	**0.556 (0.509-0.681)**	**0.039**	0.554 (0.472-0.560)	0.575 (0.556-0.598)	0.125
M_HDL_C	0.553 (0.495-0.664)	0.671 (0.612-0.824)	0.055	0.655 (0.538-0.679)	0.677 (0.669-0.689)	0.438
M_HDL_CE	0.461 (0.410-0.546)	0.561 (0.503-0.682)	0.074	0.541 (0.447-0.564)	0.558 (0.547-0.573)	0.438
M_HDL_FC	0.092 (0.085-0.118)	0.110 (0.109-0.142)	0.058	0.114 (0.091-0.115)	0.119 (0.116-0.121)	0.125
**M_HDL_TG**	**0.025 (0.014-0.033)**	**0.040 (0.013-0.042)**	**0.039**	0.024 (0.021-0.037)	0.042 (0.014-0.045)	0.313
**S_HDL_P**	**0.010 (0.009-0.011)**	**0.012 (0.011-0.013)**	**0.008**	0.011 (0.011-0.011)	0.012 (0.012-0.012)	0.125
**S_HDL_L**	**1.107 (1.027-1.222)**	**1.309 (1.254-1.417)**	**0.013**	1.261 (1.199-1.263)	1.315 (1.281-1.355)	0.063
**S_HDL_PL**	**0.623 (0.561-0.654)**	**0.730 (0.697-0.775)**	**0.012**	0.683 (0.672-0.690)	0.717 (0.708-0.736)	0.063
**S_HDL_C**	**0.462 (0.423-0.524)**	**0.567 (0.496-0.591)**	**0.008**	0.525 (0.505-0.526)	0.559 (0.542-0.561)	0.125
**S_HDL_CE**	**0.343 (0.309-0.393)**	**0.433 (0.371-0.446)**	**0.008**	0.389 (0.382-0.393)	0.412 (0.405-0.418)	0.188

**S_HDL_FC**	**0.119 (0.114-0.130)**	**0.137 (0.130-0.145)**	**0.008**	0.132 (0.122-0.137)	0.139 (0.137-0.141)	0.063
S_HDL_TG	0.031 (0.024-0.039)	0.041 (0.030-0.051)	0.098	0.034 (0.030-0.047)	0.042 (0.024-0.056)	0.588
**CRP**	**1.1 (0.81-3.38)**	**0.2 (0.06-1.12)**	**0.004**	0.24 (0.09-0.97)	0.11 (0.05-0.16)	0.313

CRP: C-reactive protein. The correlation analysis of CRP and HDL-related metabolites of different sizes of HDL. Statistically significant differences are marked in bold ($p<0.05$), and the statistical method is Spearman correlation analysis.

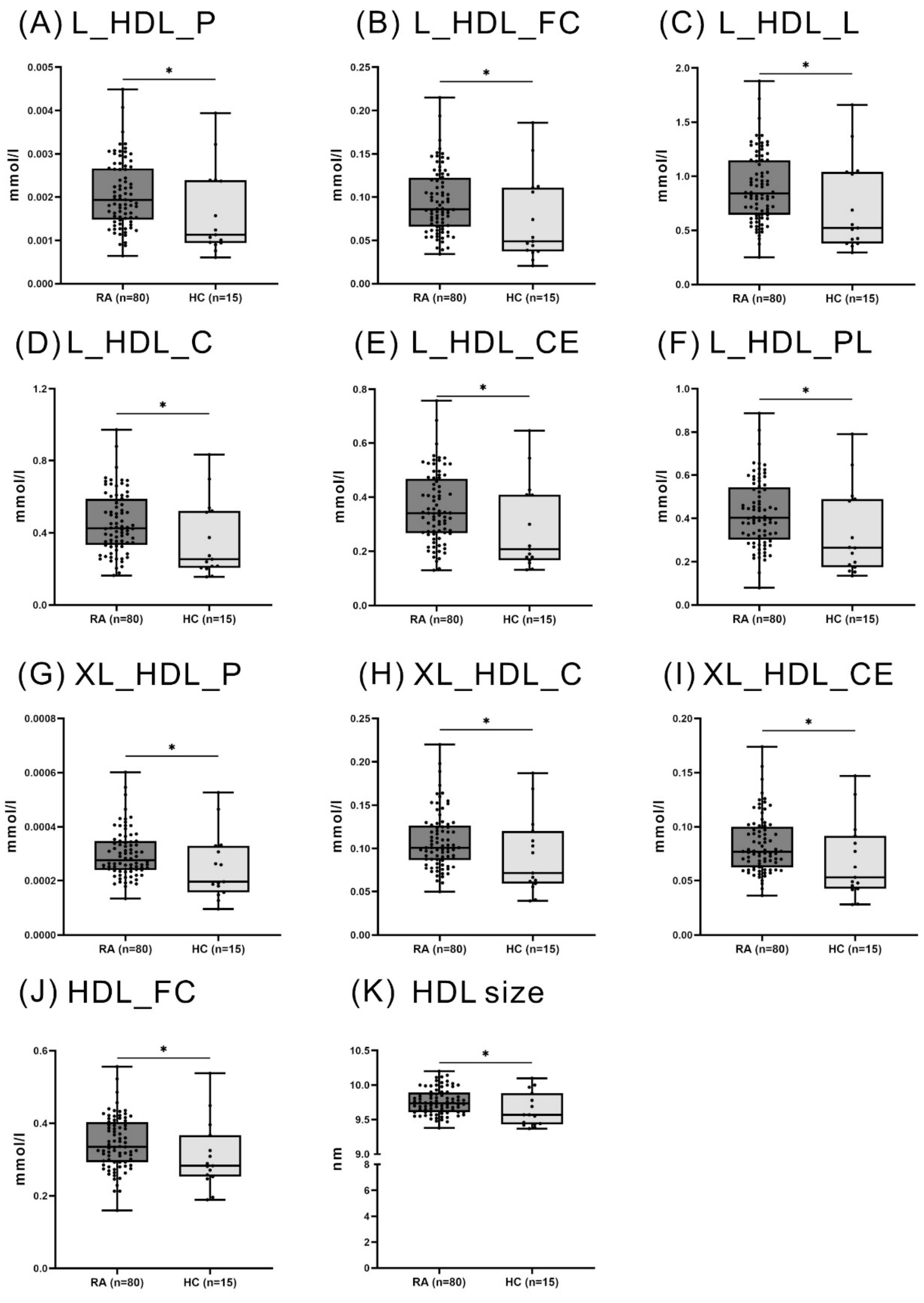

Figure S1. The difference in HDL related metabolites profile between RA and HC. * $p < 0.05$, determined by using Mann-Whitney U test.

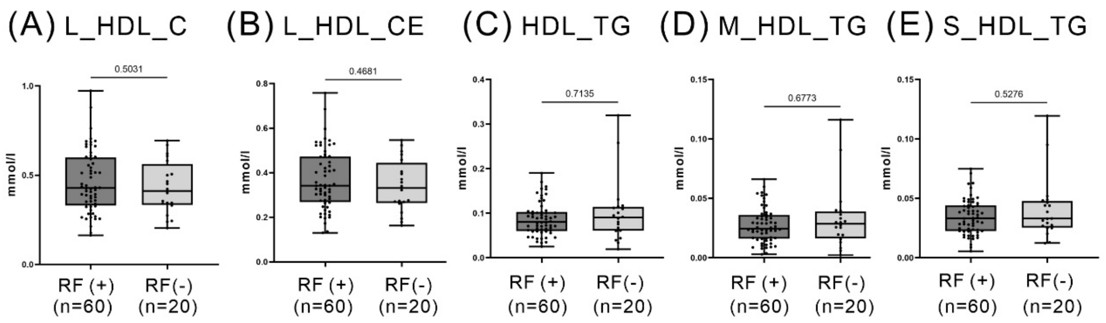

Figure S2. The difference in the levels of discriminant HDL-related metabolites between RA patients with and without RF. The difference in metabolites between patients with and without RF (A-E). * $p < 0.05$, determined by using Mann-Whitney U test.
